# Treatment failure among Sub-Sahara African children living with HIV: a systematic review and meta-analysis

**DOI:** 10.1186/s13052-024-01706-w

**Published:** 2024-10-01

**Authors:** Belete Gelaw Walle, Chalie Marew Tiruneh, Moges Wubneh, Bogale Chekole, Amare Kassaw, Yibeltal Assefa, Kelemu Abebe, Zeamanuel Anteneh Yigzaw

**Affiliations:** 1 Department of Pediatric and Child Health Nursing, School of Nursing, College of Health Science and Medicine, Wolaita Sodo University, Wolaita, Sodo, Ethiopia; 2 Department of Pediatric and Child Health Nursing, College of Medicine and Health Sciences, Debre Tabor University, Debre Tabor, Ethiopia; 3 Department of Adult health Nursing, College of Medicine and Health Sciences, Debre Tabor University, Debre Tabor, Ethiopia; 4 Department of Pediatric and Child Health Nursing, College of Medicine and Health Sciences, Wolkite University, Wolkite, Ethiopia; 5 School of Public Health, College of Health Science and Medicine, Wolaita Sodo University, Wolaita Sodo, Ethiopia; 6 School of Midwifery, College of Health Science and Medicine, Wolaita Sodo University, Wolaita Sodo, Ethiopia; 7 Department of health promotion, College of Medicine and Health Sciences, Bahir Dar University, Bahir Dar, Ethiopia

**Keywords:** HIV/AIDS, Treatment failure, Systematic review, Meta-analysis, Sub-Sahara Africa

## Abstract

**Background:**

Antiretroviral treatment failure is a global issue, particularly in developing countries such as Sub-Saharan Africa. Prior research findings were highly variable and inconsistent across areas. As a result, the goal of this systematic review and meta-analysis was to determine the pooled prevalence of treatment failure among children receiving antiretroviral medication in Sub-Saharan Africa.

**Methods:**

To find qualifying papers, we searched databases (such as PubMed, Google Scholar, African Journals Online, Scopus, and the Cochrane Library). The data were retrieved using Microsoft Excel and exported to STATA Version 14 for analysis. To check for publication bias, we employed Egger and Begg’s regression tests. A random-effects model was used to assess the pooled prevalence of treatment failure due to high levels of variability.

**Results:**

Following the removal of duplicated articles and quality screening, a total of 33 primary articles were determined to be appropriate for inclusion in the final analysis for this study. Overall, the pooled prevalence of treatment failure among HIV-infected children was 25.86% (95% CI: 21.46, 30.26). There is great variety across the included studies, with the majority of them being conducted in Ethiopia. Cameroon had the greatest pooled prevalence of treatment failure among HIV-infected children, at 39.41% (95% CI: 21.54, 57.28), while Ethiopia had the lowest, at 13.77% (95% CI: 10.08, 17.47).

**Conclusions:**

The pooled estimate prevalence of treatment failure among HIV-infected children in Sub-Saharan Africa was high. The implementation of national and international policies and strategies on ART clinic care services should be given special focus in order to reduce treatment failure in children living with HIV/AIDS.

**Trial registration:**

The protocol has been registered in the PROSPERO database under the registration number CRD-429011.

## Introduction

Acquired immune deficiency syndrome (AIDS) is a viral disease caused by the human immunodeficiency virus (HIV) that weakens the immune system and increases susceptibility to opportunistic infections [1]. The Human Immunodeficiency Virus (HIV) pandemic affects a large portion of the global population [2]. HIV has many routes of transmission including mother-to-child transmission [3]. Several activities have been implemented on prevention of mother to child transmission (PMTCT) intervention since, such as increasing institutional delivery, infant prophylaxis, Antiretroviral coverage, and proper feeding practices of infants [3–5]. In 2018, the number of people living with HIV worldwide exceeded 37.9 million.. Around 1.8 million of them were children (aged under 15 years) [6]. This year, 23.3 million HIV-positive individuals worldwide had access to antiretroviral therapy (ART) [7].

Antiretroviral therapy (ART) is critical for slowing the progression of HIV/AIDS, improving patient health, and ensuring long-term access to care for HIV-infected patients [8, 9]. Maintaining long-term ART adherence, viral load suppression, and preventing of antiretroviral therapy failure, on the other hand, remains a challenge for HIV-infected children [10]. Treatment failure can be classified as immunological, clinical, virological, or a mix of these [11].

Treatment failure can be more accurately and informatively determined by looking at virological failure, based on existing standards [11, 12]. In the absence of viral load monitoring, the move to second-line medication regimens is based on clinical criteria (i.e. opportunistic infections) or immunological criteria (patient cell differentiation CD4 T-cell count) [11]. According to the Joint United Nations Program on HIV/AIDS (UNAIDS) and partners, 90% of individuals on ART will have viral suppression by the end of 2030 worldwide [11, 13]. As a result, executing globally approved preventive measures and detecting treatment failure early is critical for treatment efficacy and meeting a stated strategic treatment goal of 2030 [14, 15].

The goal of this systematic review and meta-analysis was to estimate the rates of treatment failure among children on ART using available primary studies in Sub-Saharan Africa, which has one of the highest rates of HIV/AIDS worldwide. The estimated prevalence of treatment failure in Sub-Saharan Africa countries was inconsistent with wide ranges. In South Africa, for example, the prevalence of treatment failure ranged from 12.6% [16] to 19.3% [17], in Tanzania from 25.4% [18] to 57.1% [19], in Cameroon from 25% [20] to 53% [21], in Nigeria from 13.6% [22] to 32.9% [23], and in Ethiopia from 3.1% [24] to 22.6% [25].The findings of this study will allow countries to sustain treatment successes and hasten the decline of childhood treatment failure in the region, as well as assist decision makers and other concerned stakeholders in designing, implementing, and evaluating interventions to improve level of ART adherence.

## Materials and methods

### Study identification and reporting

We researched both published and unpublished studies regarding the prevalence of treatment failure in HIV-infected children. The results of this systematic review and meta-analyses were prepared and presented according to the Preferred Reporting Items for Systematic Reviews and Meta-analyses (PRISMA) guidelines [26].

### Study design, settings and search strategies

The pooled prevalence of treatment failure among HIV-infected children in Sub-Saharan African nations was estimated using a comprehensive review and meta-analysis. A comprehensive search was undertaken on PubMed, Google Scholar, African Journals Online, Scopus, and the Cochrane Library databases to find possibly relevant primary articles. Some internet repository library centers were explored for unpublished relevant literatures to this study. Furthermore, gray literatures have been identified through the review of reference lists and the involvement of subject matter experts. To search on the advanced PubMed search engine, MeSH (Medical Subject Headings), Boolean operators, and all fields within records were applied. Children, child, pediatrics, treatment failure, antiretroviral therapy, prevalence, proportion, and associated factors were among the search terms or phrases used.

The advanced PubMed database search strategy was performed using the following key terms.

(((((((((Prevalence[tw] OR proportion[tw])) OR ("Prevalence"[MeSH Terms] OR "magnitude"[MeSH Terms] OR "incidence"[MeSH Terms]))) AND (((Treatment failure[tw] OR immunological failure[tw] OR clinical failure [tw] OR virological failure[tw])) OR ("treatment failure"[MeSH Terms] OR "treatment"[All Fields] OR "Failures"[MeSH Terms] OR "Treatment Outcome"[MeSH Terms]))) AND (((factors[tw] OR determinants[tw] OR predictors[tw])) OR ("factors"[MeSH Terms] OR "risk factors"[MeSH Terms] OR "determinants"[MeSH Terms]))) AND (((Children[tw] OR pediatrics[tw] OR Infant[tw])) OR ("child"[MeSH Terms] OR "pediatrics"[MeSH Terms] OR "infant"[MeSH Terms]))) AND (((Antiretroviral therapy [tw] OR ART[tw])) OR (("anti-retroviral agents"[All Fields] OR "anti-retroviral agents"[MeSH Terms] OR "therapeutics"[MeSH Terms]))) AND (((Human Immunodeficiency Virus[tw] OR HIV[tw] OR AIDS[tw])) OR ("HIV"[MeSH Terms] OR "acquired immunodeficiency syndrome"[MeSH Terms]))) AND Sub-Sahara African[tw]. The search was done between April 7 and May 29, 2023. All papers published up to May 29, 2023 were included. Endnote X8 software manager was used to cite references and manage the searched literatures.

### Outcome measurements

The outcome measure of interest in this study is HIV/AIDS treatment failure, which was defined by the WHO as immunological, clinical, and virological treatment failure [11].

### Eligibility criteria

#### Inclusion criteria

##### Study area

Only studies conducted in sub-Saran Africa were included to produce single estimate of common effects.

##### Study design

All observational study designs reporting the prevalence of treatment failure were eligible for this meta-analysis.

##### Population

All HIV-infected children on antiretroviral treatment.

##### Language

Only articles reported in English language were incorporated.

##### Publication condition

Both published and unpublished studies were considered.

#### Exclusion criteria

We omitted primary studies that did not provide quantifiable treatment failure outcomes for children or did not pass our quality screening. Conference reports and papers that did not provide access to the full text were excluded. These researches were excluded due to the inability to evaluate the quality of papers in the absence of complete text.

### Data selection process

Three authors (BG, KA, and BC) retrieved the relevant data from the included articles using a standardized data extraction format adapted from the Joanna Briggs Institute (JBI). Duplicate articles and articles with titles indicating that they did not deal with antiretroviral treatment failure were removed. The three reviewers additionally evaluated every article against the inclusion and exclusion criteria. Any differences during screening were resolved through dialogue. The data selection form comprised the first author’s name, publication year, country of study, study area, study design, sample size, response rate, and prevalence with 95% CI.

### Quality assessment

The whole text of the articles was assessed for relevance based on titles, objectives, and method, and the quality of the included articles was rated by four investigators (BGW, CMT, AK, and YA) using the Newcastle–Ottawa Scale quality evaluation tool for observational studies [27]. Any disagreements between the four quality assessors were handled by repeating the procedures and involving a third reviewer before computing the final appraisal results. The Newcastle–Ottawa Scale scores of four independent reviewers were averaged to determine the quality of included articles.

### Data processing and statistical analysis

The required data were retrieved from the primary articles using Microsoft Excel and imported to STATA software version 14 for further analysis. The random-effects model developed by DerSimonian and Laird was used to assess the overall pooled prevalence of treatment failure [28]. The *p*-values of the Cochrane Q and I^2^ test statistics [29] were used to determine heterogeneity. Subgroup analysis was performed to account for random variation in the original study’s point estimations and to analyze how failure varies across subgroup participants. Sensitivity analysis was used to look for outliers among the collected articles. Publication bias across studies was assessed using funnel plot and egger’s regression test. The Egger’s regression test results were not statistically significant for publication bias at the 5% significance level [30]. A forest plot format was used to present the point prevalence and 95% CIs. In this plot, the weight of study was indicated by the size of each box, while each crossed line referred to a 95% confidence interval. The effect size estimates were reported in the form of pooled prevalence.

## Results

### Study selection

**Fig. 1:**
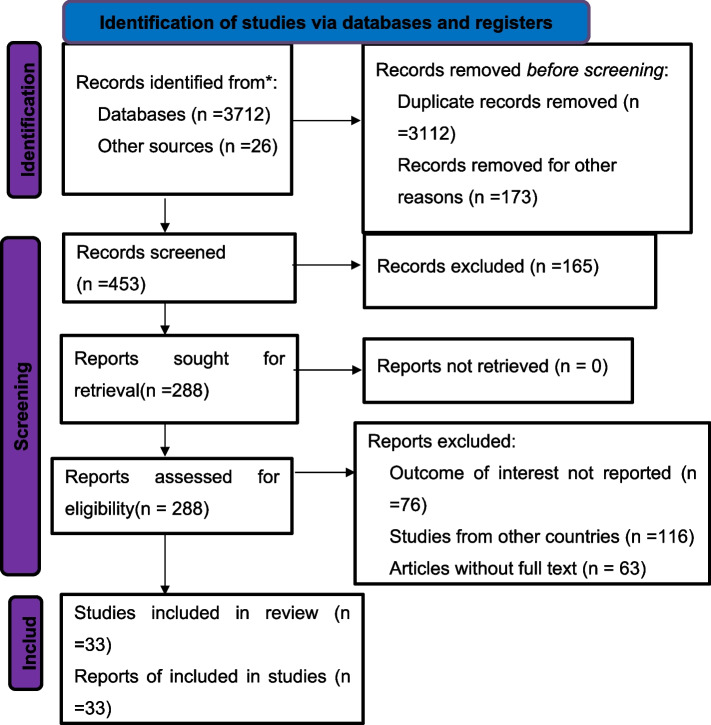
PRISMA 2020 flow chart of primary study selection for systematic review and meta-analysis of treatment failure among HIV infected children in Sub-Sahara Africa, 2009 to 2023

We retrieved 3,738 articles from PubMed, Google Scholar, African Journals Online, Cochrane Library, Scopus, and other sources. There were approximately 626 non-duplicated articles among the initial articles. After reviewing the titles and abstracts of the remaining articles, 228 were excluded. Then, 225 potentially full-text articles were evaluated for eligibility using pre-defined criteria, and 192 articles were dropped for various reasons. Finally, 33 articles satisfied the inclusion criteria and were included in the final meta-analysis to assess the prevalence of treatment failure (Fig. 1).

### Characteristics of included studies

This study comprised 33 original publications with a total of 21,722 study participants published between 2009 and 2023 from various parts of Sub-Saharan African countries. Of the 33 included primary articles, 12 were from Ethiopia [24, 25, 31–40], 4 were from Tanzania [18, 19, 41, 42], 3 were from Cameroon [20, 21, 43], 3 were from Uganda [44–46], 3 were from Nigeria [22, 23, 47], 3 were from South Africa [16, 17, 48], and the remaining 5 were from Eritrea [49], Kenya [50], Malawi [51], Senegal [52], and Zimbabwe [53]. Twenty-two of the studies were cohort studies [16, 17, 22–25, 31–36, 38, 40, 43–49, 51], ten were cross-sectional studies [18–21, 37, 39, 41, 42, 50, 52, 53], and one was a case–control study design [50] (Table 1). The sample size of individual articles in our study ranged from 35 [51] to 5485 [17].

**Table 1:** General characteristics of studies included in systematic review and meta-analysis of treatment failure among HIV-infected children in Sub-Sahara Africa, 2009–2023

Authors	Study design	Publication year	Country	Sample	Prevalence(%)	Quality assessment
Zoufaly etal	Cross-sectional	2013	Cameroon	230	53	Good
Nlend etal	Cross-sectional	2016	Cameroon	375	25	Good
Ngoupo etal	Cohort	2021	Cameroon	155	40.6	Good
Mengstu etal	Cohort	2023	Eritrea	724	38.5	Good
Getawa et al	Cross-sectional	2021	Ethiopia	200	12.5	Good
Haile et al	Cohort	2019	Ethiopia	391	22.6	Good
Yihun et al	Cohort	2019	Ethiopia	402	12.19	Good
Gelaw et al	Cross-sectional	2021	Ethiopia	424	14.8	Good
Tadesse et al	Cohort	2017	Ethiopia	628	3.1	Good
Netsanet et al	Cohort	2009	Ethiopia	96	11.5	Good
Yassin et al	Cohort	2017	Ethiopia	269	18.8	Good
Sisay et al	Cohort	2018	Ethiopia	824	7.7	Good
Bacha et al	Cohort	2012	Ethiopia	1,186	14.1	Good
Zeleke et al	Cohort	2016	Ethiopia	225	18.2	Good
Getaneh etal	Cohort		Ethiopia	554	17.3	Good
Kadima etal	Case–control	2018	Kenya	1190	37	Good
Huibers et al	Cohort	2018	Malawi	35	66	Good
Orji et al	Cohort	2021	Nigeria	88	13.6	Good
Ebonyi etal	Cohort	2014	Nigeria	580	18.5	Good
Boerma etal	Cohort	2016	Nigeria	82	32.9	Good
Cisse et al	Cross-sectional	2019	Senegal	851	64	Good
Davies et al	Cohort	2012	South Africa	5485	19.3	Good
Meyers et al	Cohort	2015	South Africa	1692	12.6	Good
Bitwale etal	Cross-sectional	2021	Tanzania	300	34	Good
Mgelea etal	Cross-sectional	2014	Tanzania	218	57.1	Good
Emmett et a	Cross-sectional	2010	Tanzania	206	31.6	Good
Sebunya et al	Cohort	2013	Ugandan	701	34	Good
Costenaro et al	Cohort	2014	Mozambique and Uganda	740	29	Good
Boerma etal	Cohort	2017	Ugandan	64	20	Good
Makadzange etal	Cross-sectional	2015	Zimbabwe	599	30.6	Good
Muri et al	Cross-sectional	2017	Tanzania	213	25.4	Good
Wondifraw et al	Cohort	2022	Ethiopia	336	14	Good
Makatini etal	Cohort	2021	South Africa	1659	18.5	Good

### Results of individual studies

#### Risk of bias in studies

**Fig. 2:**
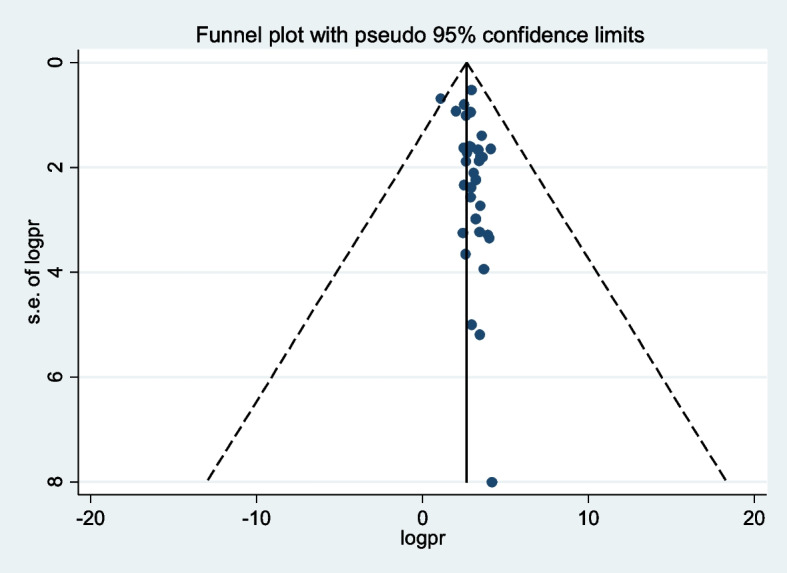
Funnel plot with 95% confidence limits of the pooled prevalence of treatment failure among HIV-infected children in Sub-Sahara Africa, 2009 to 2023

The Egger’s test was used to check for publication bias, which revealed no statistically significant publication bias with a *p*-value of 0.78. We also used a funnel plot to assess publication bias for overall treatment failure (Fig. 2).

#### Heterogeneity and sensitivity analysis

The heterogeneity test (I^2^) result was 98.6%, *p* < 0.01, indicating that there is significant variety across the included primary articles. In the sensitivity analysis, all studies were within the confidence interval, and no single study contributed to publication bias.

#### Prevalence of HIV/AIDS treatment failure

**Fig. 3:**
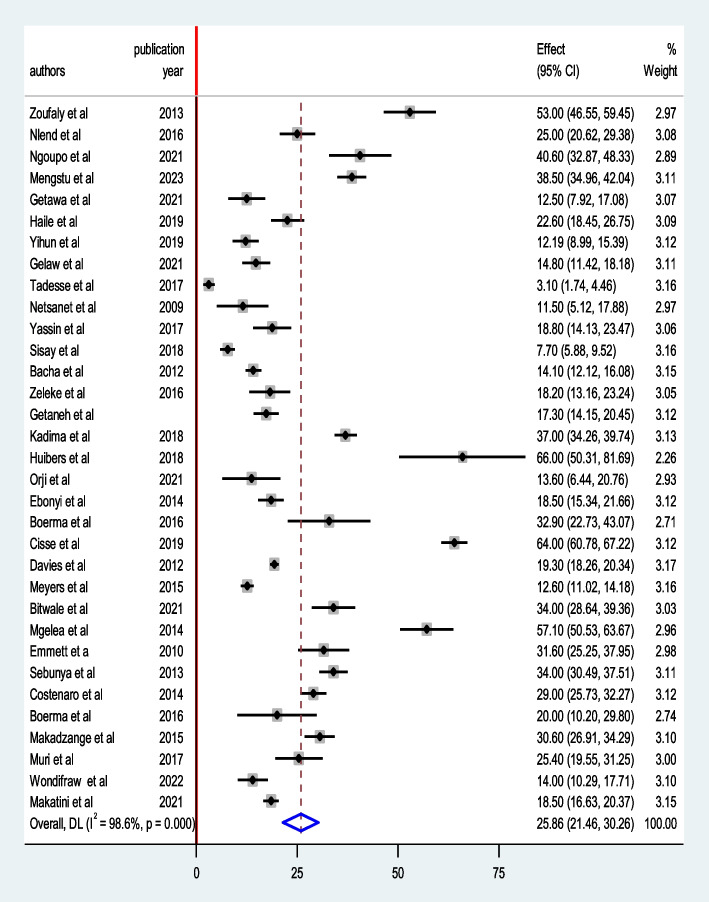
Forest plot of the pooled prevalence of treatment failure among HIV- infected children in Sub-Sahara Africa, 2009 to 2023

The overall pooled prevalence of treatment failure among HIV-infected children in Sub-Saharan Africa was found to be 25.86% (95% CI: 21.46, 30.26, I^2^ = 98.6%, *P* < 0.01) (Fig. 3).

### Subgroup analysis

Various criteria were used to assess the subgroup prevalence of treatment failure. These were the geographical settings, study design type, and publication year. Our subgroup analyses revealed that the largest proportion of treatment failure was seen among HIV-infected children residing in Cameroon (39.41% (95% CI: 21.54, 57.28, I^2^ = 98.1%, *P* < 0.01) and the lowest in Ethiopia (13.77% (95% CI: 10.08, 17.47, I^2^ = 94.9%, *P* < 0.01) (Table 2). Treatment failure among HIV-infected children was 34.76% (95% CI: 22.44, 47.06, I^2^ = 98.6%, *P* < 0.01), and 20.82% (95% CI: 17.02, 24.62, I^2^ = 97.7%, *P* < 0.01) in cross-sectional and cohort studies, respectively (Table 2). Furthermore, the prevalence of treatment failure among HIV-infected children was 27.87% (95% CI: 22.30, 33.45, I^2^ = 97.9%, *P* < 0.01) in studies published from 2009 to 2015, yet it was 25.29% (95% CI: 18.25, 32.33, I^2^ = 98.9%, *P* < 0.01) in articles published from 2016 to 2023 (Table 2).

**Table 2:** Summary of subgroup analysis for the pooled prevalence of treatment failure among HIV-infected children in Sub-Sahara Africa, 2009 to 2023 (*n* = 33)

Variables	Subgroup	Prevalence,% (95% CI, I^2^, *P*-value)
Country	Cameroon	39.41 (21.54–57.28, 96.10, < 0.01)
	Ethiopia	13.77 (10.08–17.47, 94.9, < 0.01)
	Nigeria	20.66 (12.44–28.88, 78.80, 0.009)
	South Africa	16.81 (12.59–21.03, 96, < 0.01)
	Tanzania	36.96 (24.15–49.77, 94.50, < 0.01)
	Other	39.30 ( 29.91–48.69, 97.80, < 0.01)
Study design	Cross-sectional	34.76 (22.44–47.08, 98.60, < 0.01)
	Cohort	20.82 (17.02–24.62, 97.70, < 0.01)
Publication year	2009–2015	27.87 (22.30–33.45, 97.90, < 0.01)
	2016–2023	25.29 (18.25–32.33, 98.90, < 0.01)

## Discussion

Despite advancements in comprehensive HIV care facilities and ART coverage, HIV/AIDS transmission and the rate of treatment failure among HIV-infected children remain a global problem, particularly in Sub-Saharan African countries. Treatment failure is one of the leading causes of childhood morbidity and mortality in resource limited countries. This meta-analysis and systemic review was conducted to determine the pooled prevalence of treatment failure among HIV-infected children in Sub-Saharan African countries. The findings will help patients stay on first-line ART regimens longer, avoiding the more expensive and dangerous second-line ART regimens in developing countries.

The pooled prevalence of treatment failure among HIV-infected children in Sub-Saharan African countries was 25.86% (95% CI: 21.46, 30.26), as per the findings of this meta-analysis. Our findings are consistent with previous research on HIV-infected children in Mozambique and Uganda (29%) [45], Tanzania (25.4%) [18], Cameroon (25%) [20], and Ethiopia (22.6%) [25]. This suggests that simply initiating ART for HIV-infected children is insufficient, and that treatment failure prevention initiatives, regular therapeutic medication monitoring, and resistance testing in the area should be prioritized instead. A higher burden of HIV/AIDS treatment failure may be associated with lower socioeconomic level indices in Sub-Saharan African nations (such as unemployment, lack of education beyond a university, financial difficulties, and rental or unstable housing status).

Our finding is significantly greater than the findings of the other meta-analyses and observational studies. For example, the prevalence of treatment failure was found in Ethiopia (12.34%) [54], South Africa (12.6%) [16], Nigeria (13.6%) [22], and Uganda (20%) [46] based on prior studies. This figure, on the other hand, is significantly lower than studies conducted in Malawi (66%) [51], Tanzania (57.1%) [19], Eritrea (38.5%) [49], Kenya (37%) [50], Senegal (64%) [52], and Cameroon (53%) [21]. The aforementioned disparities could be attributed to differences in study technique and sample size utilized to diagnose treatment failure by individual studies conducted in each country. This could be due to variation in patient monitoring standards or the quality of medical services, socioeconomic status, and an increase in the number of patients on ART over time, all of which could have an impact on the capacity of HIV treatment failure diagnosis, monitoring, adherence, and treatment outcome in general.

There is statistically significant heterogeneity among the included primary studies in this systematic review and meta-analysis. Thus, we conducted subgroup analysis. As a result, Ethiopia had the lowest prevalence of HIV treatment failure (13.77% (95% CI: 10.08, 17.47) among Sub-Saharan African countries. Furthermore, Cameroon had the highest rate of treatment failure among HIV-infected Sub-Saharan African children, at 39.41% (95% CI: 21.54, 57.28). The disparity may be attributed to differences in study design, sample size, and the number of primary studies included in our review from each country.

In Ethiopia, HIV/AIDS patients treatment is decentralized, with the majority of ART services delivered at the primary health care facility level [55]. Additionally, health systems in countries such as Ethiopia may have an impact on the quality of care, patient monitoring, and treatment adherence [56]. In Ethiopia’s healthcare system, skilled health extension workers provide follow-up care to HIV/AIDS patients in the community, especially rural areas. Health extension workers provide health information on HIV/AIDS and other community health issues [57]. This may assist to reduce stigma and discrimination in the community, which may improve ART adherence and treatment outcomes.

In Sub-Saharan African countries, the burden of treatment failure among HIV-infected children remains significant. This could be correlated to ART delay [58], opportunistic infections [59], poor HIV care, late identification of HIV treatment failure [60], ART adverse response [61], dietary issues [62], and low ART adherence [63]. As a result, HIV-infected children require special attention during their ART follow-up visit because they are more prone to have treatment failure.

### Limitations of the review

Many limits must be considered before interpreting the results of this review. This review covered only articles written in English, which may exclude some works. In addition, the majority of studies included in this investigation were from Ethiopia, resulting in a significant degree of variability that limits our ability to determine the precise prevalence of treatment failure across the country. Furthermore, because this meta-analysis covers a limited number of trials, the results may not accurately represent regional failure rates.

## Conclusion

This meta-analysis found that the prevalence of treatment failure among HIV-infected children in Sub-Saharan African nations remained significantly high. It is suggested that children living with HIV require additional treatment adherence counseling, care, and support. Treatment failure assessment and interventions should be prioritized during HIV care services for children at ART clinics in each country.

## Data Availability

All generated or analyzed data during this study will be available from the supporting information file.

## Abbreviations

AIDS: Acquired immune deficiency syndrome
ART: Antiretroviral therapy
CI: Confidence interval
HIV: Human immunodeficiency virus
WHO: World health organization
